# Visuospatial Attention and Saccadic Inhibitory Control in Children With Cerebral Palsy

**DOI:** 10.3389/fnhum.2019.00392

**Published:** 2019-11-08

**Authors:** Claudio Maioli, Luca Falciati, Jessica Galli, Serena Micheletti, Luisa Turetti, Michela Balconi, Elisa M. Fazzi

**Affiliations:** ^1^Department of Clinical and Experimental Sciences, University of Brescia, Brescia, Italy; ^2^Unit of Child Neurology and Psychiatry, ASST Spedali Civili of Brescia, Brescia, Italy; ^3^Research Unit in Affective and Social Neuroscience, Department of Psychology, Catholic University of Milan, Milan, Italy

**Keywords:** cerebral palsy, eye movements, inhibitory control, executive skills, visuospatial attention, saccades, oculomotor control, cueing paradigm

## Abstract

Cerebral palsy (CP) is a non-progressive syndrome due to a pre-, peri- or post-natal brain injury, which frequently involves an impairment of non-motor abilities. The aim of this article was to examine visuospatial attention and inhibitory control of prepotent motor responses in children with CP showing a normal IQ or mild cognitive impairment, measuring their performance in oculomotor tasks. Ten children (9–16-year-old) with spastic CP and 13 age-matched, typically developing children (TDC) participated in the study. Subjects performed a simple visually-guided saccade task and a *cue-target* task, in which they performed a saccade towards a peripheral target, after a non-informative visual cue was flashed 150 ms before the imperative target, either at the same (*valid*) or at a different (*invalid*) spatial position. Children with CP showed severe executive deficits in maintaining sustained attention and complying with task instructions. Furthermore, saccadic inhibitory control appeared to be significantly impaired in the presence of both stimulus-driven and goal-directed captures of attention. In fact, patients showed great difficulties in suppressing saccades not only to the cue stimuli but also to the always-present target placeholders, which represented powerful attentional attractors that had to be covertly attended throughout the task execution. Moreover, impairment did not affect in equal manner the whole visual field but showed a marked spatial selectivity in each individual subject. Saccade latencies in the *cue-target* task were faster in the *valid* than in the *invalid* condition in both child groups, indicating the preservation of low-level visuospatial attentive capabilities. Finally, this study provides evidence that these impairments of executive skills and in inhibitory control, following early brain injuries, manifest in childhood but recover to virtually normal level during adolescence.

## Introduction

Cerebral palsy (CP) designates a group of non-progressive neurological disorders, because of a pre-, peri- or post-natal brain injury, affecting the development of movement and postural abilities (Bax et al., 2005; Rosenbaum et al., 2007). However, cerebral damage in this neurodevelopmental condition is in general not restricted to the motor system. Children with CP (CPC) frequently manifest a varying degree of neurovisual, cognitive and learning deficits (Rosenbaum et al., 2007; Fazzi et al., 2009, 2012; MacLennan et al., 2015). In fact, lesions of the periventricular white matter and of the cortical deep gray matter are very common in CP, leading to the involvement not only of motor abilities, but also of non-motor developing abilities, such as visuospatial, attentive and executive functions (Krägeloh-Mann and Horber, 2007; Galli et al., 2018).

Severity of non-motor symptoms varies substantially in CP. However, even children with cognitive functions within or above the normal range manifest a higher prevalence of learning disorders (Frampton et al., 1998) and dysfunctions in sustained and divided attention (Kolk and Talvik, 2000; Pirila et al., 2004; Bottcher et al., 2010). These dysfunctions are associated with increased distractibility and inattention and may explain why CPC have often a lower academic performance and problems in emotional and social relationships (Nadeau and Tessier, 2006; Parkes et al., 2009; Whittingham et al., 2010). Visuospatial attention plays a pivotal role as a filter mechanism for selecting which parts of the visual scene are relevant in a given behavioral context. In a limited-capacity system, such as that of the brain in processing an enormous amount of information, the ability of selecting relevant stimuli from background is not only essential for sensorimotor integration but is also critical for the development of executive and academic skills (Anderson, 2002). Furthermore, a causal link has been proposed between visuospatial attention and reading acquisition (Hari and Renvall, 2001; Vidyasagar and Pammer, 2010; Franceschini et al., 2012; Collis et al., 2013). In view of the high incidence of reading difficulties in CPC (Frampton et al., 1998; Schenker et al., 2005; Gillies et al., 2018), deficits in selective attention may be responsible also for learning disorders in CP (Bottcher, 2010).

Attention and executive dysfunctions have been mostly ascertained in CP by means of neuropsychological tests and questionnaires (e.g., White and Christ, 2005; Bottcher et al., 2010; Bodimeade et al., 2013; Whittingham et al., 2014). Only few articles investigated visual attention in children with spastic diplegic CP by using an orienting task (Craft et al., 1994; Schatz et al., 2001). These studies employed a reaction-time paradigm, developed by Posner and colleagues, in which a manual response follows a covert orientation of attention to a peripheral visual target (i.e., maintaining gaze at a central fixation point), after presenting a visual cue at the same or at a different spatial location (Posner, 1980; Posner et al., 1985). Both reports described a pattern of impairments in basic attentional mechanisms, which was associated with a prevalent damage of anterior brain regions, suggesting that frontal cortical areas play a critical role in the development of visual attention. Since frontal lobes are well known to play an important role also in the developing of executive abilities (Stuss et al., 1997; Casey, 2001), one could expect that attentive and executive impairments coexist in CPC.

The evaluation of oculomotor functions represents in many aspects an ideal tool to investigate at once both selective attention and the competence of executive abilities. Specifically, in this article we adopted a saccadic task, similar to the Posner cuing protocol, in which however the participant must perform an eye movement towards the peripheral target after a non-informative visual cue is flashed either at the same or at a different spatial position. By monitoring only the eye movements, the limitations in posture and limb movements that characterize CP become irrelevant in determining the accuracy and the timing of the motor response. In fact, in the absence of strabismus, nystagmus or ocular motor apraxia (Lanzi et al., 1998; Jacobson and Dutton, 2000), the saccadic system of most CPC does not differ, or shows only very modest abnormalities, with respect to typically developing children (TDC; Katayama and Tamas, 1987; Christ et al., 2003; Saavedra et al., 2009).

The correct execution of the oculomotor task adopted in this article requires a proper operation of a number of cognitive abilities. First, the task requires the capacity of actively keeping a steady fixation, maintaining sustained attention for a long time at a specific point of the visual scene. Second, selective attention must identify the sensory event that is relevant to the immediate goal, discarding a distracting cue that is irrelevant to the task. Third, inhibitory mechanisms must suppress unwanted motor responses. The abrupt onset of the cue in the perceptual space elicits an automatic, bottom-up selection process for action, even if it is in contrast with the prescriptions of the task. The suppression of this motor response requires the activation of inhibitory mechanisms by the prefrontal executive system, possibly with the involvement of frontostriatal circuits (Casey, 2001). Finally, if a visual cue attracts attention at the same spatial location of the target, shortly before its onset, a facilitation effect determines an increase of the response speed. In TDC, as in normal adults, a cuing paradigm of this kind induces faster reaction times for both manual and saccadic responses (Posner et al., 1984; Maylor, 1985; Briand et al., 2000).

The aim of this article is to examine visuospatial attention and executive abilities of CPC, measuring their performance in an oculomotor task. We employed a cue-target paradigm for a quantitative evaluation of the ability to discard distracting, task-irrelevant stimuli, of engaging attentive resources for a long-lasting time span and of the capacity of inhibiting an unwanted, prepotent motor response, which is in contrast with the behavioral goal. It can be surmised that accessing the integrity of these basic skills in CP, even in children with a mild degree of disability, could be essential in view of their relevance for the development of a large number of cognitive functions. This knowledge may also address more properly therapeutic and rehabilitation approaches in order to early detect or treat learning disabilities and social difficulties, which frequently affect children with CP and adolescents (Frampton et al., 1998; Bottcher et al., 2010; Whittingham et al., 2014).

## Materials and Methods

### Ethical Approval

This study was conducted in accordance with the ethical guidelines set forth by the Declaration of Helsinki and had the approval from the Ethics Committee of “ASST Spedali Civili” of Brescia, Italy (protocol. N. 1324, 08/04/2013). Informed consent was obtained both in verbal form from the participants, as well as in written form from their parents, prior to the experimental sessions.

### Subjects

Ten children with CP (five males and five females) and 13 age-matched children (six males and seven females) with typical development (TD) participated in the study. All participants were naïve to the purpose of the experiment. CPC were enrolled in the Unit of Child Neurology and Psychiatry, at the “ASST Spedali Civili” of Brescia (Italy) and were aged from 8 years and 11 months to 16 years and 1 month (mean age: 11 years and 4 months ± 2 years and 10 months). TDC were aged from 9 years and 6 months to 15 years and 7 months (mean age: 13 years and 1 month ± 2 years and 6 months) and had no history of head trauma, neurological or psychiatric diseases, cognitive disabilities or oculomotor/neurovisual impairments.

The inclusion criteria for CPC were as follows: (1) diagnosis of spastic CP documented by neurological examination and neuroimaging according to the International Classification of CP (Bax et al., 2005; Rosenbaum et al., 2007); (2) normal IQ or mild cognitive impairment (full-scale intelligence quotient, FIQ, >50 standard scores and verbal intelligence quotient, VIQ, >70 standard scores), according to WISC-III scores (Wechsler, 2006), performed within last 12 months (see Table 1); (3) normal or near-normal visual acuity (not less than 6/10 in binocular vision); and (4) ability to understand the verbal instructions for executing the experimental task. Exclusion criteria were a history of uncontrolled epilepsy seizures and the presence of oculomotor disturbances such as nystagmus, strabismus or oculomotor apraxia (Fazzi et al., 2012).

**Table 1:** Demographic characteristics of Children with cerebral palsy (CPC).

Participant code	Handedness	GA (weeks)	CP type (Hagberg)	Motor abnormalities: nature and typology	Motor abnormalities: functional motor abilities	Associted impairments: epilepsy	Visual acuity right eye	Visual acuity left eye	FIQ	VIQ	Brain images	Brain injurycode causation and timing
CP1	Left	39	Right hemiplegia	Unilateral spastic hypertonia	GMFCS: 1 MACS: 2	focal epilepsy	10/10	10/10	83	93	Multicystic encephalopathy (left parietal-occipital-temporal areas; MRI)	Chronic circulatory insufficiency
CP2	Left	n.a.	Right hemiplegia	Unilateral spastic hypertonia	GMFCS: 1 MACS: 1	no	10/10	10/10	99	112	Bilateral Periventricular leukomalacia (MRI)	n.a.
CP3	Right	40	Left hemiplegia	Unilateral spastic hypertonia	GMFCS: 1 MACS: 3	focal epilepsy	8/10	8/10	70	63	Right porencephaly (MRI)	Prenatal
CP4	Left	40	Right hemiplegia	Unilateral spastic hypertonia	GMFCS: 2 MACS: 3	focal epilepsy	10/10	10/10	55	77	Left periventricular leukomalacia (MRI)	Hypoxic-ischemeic damage; perinatal
CP5	Left	41	Right hemiplegia	Unilateral spastic hypertonia	GMFCS: 2 MACS:2	no	10/10	10/10	67	86	Left cortical-subcortical frontal-temporal-parietal encephalomalacia (MRI)	Stroke; perinatal
CP6	Right	29	Diplegia (left > right)	Bilateral spastic hypertonia	GMFCS: 2 MACS: 1	focal epilepsy	10/10	10/10	82	89	Bilateral Periventricular leukomalacia (MRI)	Hypoxic-ischemeic damage; perinatal
CP7	Left	37	Right hemiplegia	Unilateral spastic hypertonia	GMFCS: 1 MACS: 2	no	6.3/10	6.3/10	99	92	Left periventricular leukomalacia (MRI)	Hypoxic-ischemeic damage; perinatal
CP8	Right	31	Diplegia (left > right)	Bilateral spastic hypertonia	GMFCS: 2 MACS: 1	no	9/10	9/10	87	99	Bilateral Periventricular leukomalacia (MRI)	Hypoxic-ischemeic damage; perinatal
CP9	Left	40	Right hemiplegia	Unilateral spastic hypertonia	GMFCS: 2 MACS: 2	no	10/10	10/10	99	101	Bilateral basal ganglia (putamen, thalamus) hyperintensity (MRI)	Hypoxic-ischemeic damage; perinatal
CP10	Right	29	Diplegia (left > right)	Bilateral spastic hypertonia	GMFCS: 1 MACS: 2	no	10/10	10/10	100	104	Right periventricular leukomalacia (CUS)	Hypoxic-ischemeic damage; perinatal

*CUS, cranial ultrasound; FIQ, Full-scale intelligence quotient; GA (weeks), Gestational Age (weeks); GMFCS, Gross Motor Function Classification System; MACS, Manual Ability Classification System; MRI, Magnetic Resonance Imaging; n.a., not available; VIQ, verbal intelligence quotient*.

Table 1 reports the demographic characteristics of the recruited CPC. Three CPC (CP3, CP8, CP10) were excluded from the study because of problems arising during the experimental session. Two children were unable to keep a sufficiently stable head posture on the head-support device. Consequently, it has been impossible to obtain a workable calibration for the remote eye-tracker. The third child manifested a latent strabismus during the recording session. Therefore, the average measure of the binocular gaze point showed a quite erratic behavior along the horizontal axis, yielding only very few trials with an apparently normal eye convergence. Therefore, the CPC group was composed by seven participants.

A visual field test was performed for both eyes in all CPC. The examination yielded a normal visual field in five subjects (CP2, CP4, CP6, CP7, CP9). By contrast, a visual defect was found in the lower right quadrant in CP5 and in the right hemifield in CP1. However, in these children only the peripheral vision was affected, sparing the central part of the visual field for at least 10° around the fovea. Since stimuli were presented at an eccentricity of 7°, the detected visual defects were not such to interfere with the execution of the oculomotor tasks of this study. In any case, we carefully checked, before running the experimental sessions, that all children could clearly see without effort the stimuli while looking at a fixation cross placed in the center of the visual field.

### Apparatus and Stimuli

**Figure 1:**
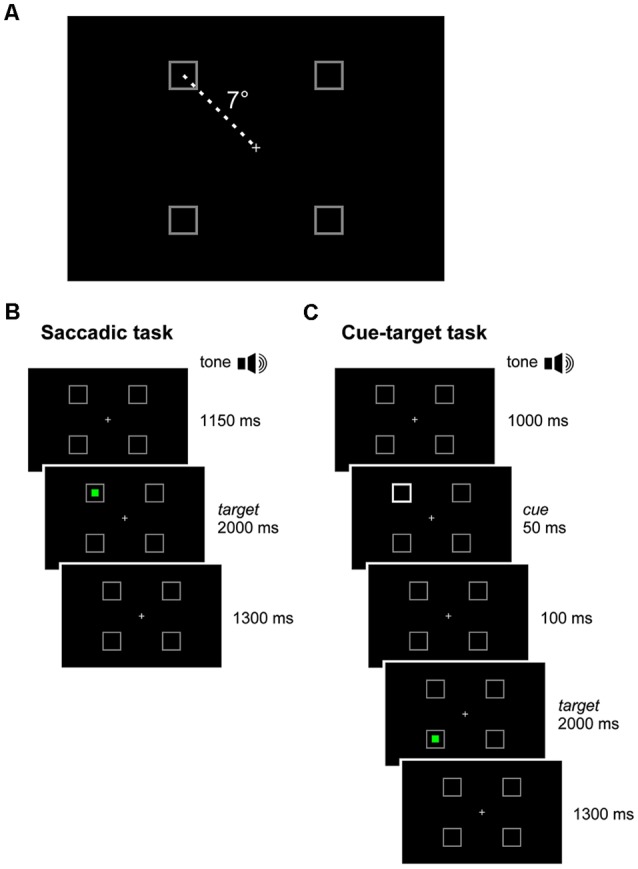
Experimental paradigm. **(A)** In each quadrant of the visual field, a square-shaped gray placeholder was displayed at 7° of eccentricity from a central cross throughout the recording session. **(B)** *Saccadic task*: the subject executed a visually-guided saccade towards a green target, randomly occurring inside one of the placeholders. **(C)** *Cue-target task*: the subject executed a saccade to the green target, disregarding a non-informative cue (50 ms luminance increase of a placeholder), which occurred 150 ms before target onset, either at the same (valid-cue condition) or at a different (invalid-cue condition) spatial location.

Participants sat in a dimly illuminated and quiet room. A combination of chin rest and head-support device was used to restrain head movements. Visual stimuli were displayed on a full HD 21.5” LED monitor (ASUS VH226H, Taiwan), located 80 cm in front of the subject. A light gray central fixation cross and four light gray square frames were displayed on the screen throughout the experimental session, against a black background. The cross subtended a visual angle of 0.36°. By contrast, the frames subtended an angle of 1.64° and served as placeholders for the visual targets. Each placeholder was located at one vertex of an imaginary square surrounding the central cross, at an eccentricity of 7° (Figure 1A).

Visual stimuli acting as saccade targets consisted of a 0.43° green solid square appearing at the center of one placeholder. Eye-movements were recorded by the remote eye-tracker Tobii X120 (Tobii AB, Stockholm, Sweden), at a sampling rate of 120 Hz and with an accuracy of 0.5°. Tobii’s image-processing algorithm, based on the reflection pattern of near-infrared light on the eyes, provided the X-Y coordinates of the gaze point on the screen in pixels, by averaging the values computed from the left and the right eyes.

The experiment was performed using Presentation^®^ software (Version 16.3, Neurobehavioral Systems, Inc., Berkeley, CA, USA^1^) and the TobiiEyetrackerExtension v1.1 for interfacing the eye tracker with the Presentation software^2^.

### Experimental Procedures

In an experimental session, participants had to perform two tasks in a sequence. The first task, hereinafter called *saccadic task* (Figure 1B), consisted in performing simple visually-guided saccades. The trial began with the central fixation cross and the four placeholders displayed on the screen. After a fixed delay of 1,150 ms from a warning tone, a green square target turned on inside one of the placeholders for 2,000 ms. The target onset was the go-signal for shifting the gaze to it, as quickly as possible. After the target disappearance, only the fixation cross and the empty placeholders remained on the visual scene for the other 1,300 ms. Afterward, a new warning tone marked the beginning of the next trial. Participants were instructed to quickly return with their gaze to the central fixation cross only after the offset of the green saccadic target. The experimental block comprised 40 trials, allowing 10 repetitions at random of each possible target location.

The second task consisted in a cuing paradigm, hereinafter called *cue-target task* (Figure 1C). This paradigm was identical to that of the *saccadic task*, except for the appearance of a visual cue 150 ms before the onset of the saccade target. The cue was represented by the doubling the luminance of one placeholder for 50 ms. The cue could occur either at the same (*valid*) or at a different (*invalid*) spatial location with respect to the saccade target. The participant had to disregard the visual cue and make, as fast as possible, an eye movement only to the green target. The cue was very little informative about the position of the forthcoming saccadic target, as it occurred at the same placeholder where the target was presented in only 40% of the trials. It is fair to assume that the small preponderance of valid cues, with respect to a completely random distribution among the placeholders, did not represent a reliable predictor of the direction of the saccadic response. In the remaining 60% of invalid-cue trials, the cue occurred randomly at one of the three placeholders with a different spatial location from the saccade target. The aim of this experimental design was to reduce the difference in sample size between valid- and invalid-cue trials. Within each experimental session, we presented 80 valid-cue trials (equally distributed among the four placeholders) and 120 invalid-cue trials (with all possible cue-target combinations occurring with equal probability), yielding a total number of 200 trials. To reduce the level of fatigue and to maintain high level of attention, we divided the *cue-target task* into five blocks of 40 trials (each one lasting about 3 min), leaving a rest period of 5 min between blocks.

All subjects received practice trials of both tasks before performing the experimental session. The calibration of the eye tracker was repeated before each block, by using a five-point routine. A correct comprehension of task instructions was carefully ascertained verbally from each child. Unfortunately, some children were unable to complete all five blocks of trials in the *cue-target task*, by manifesting restlessness in the last part of the recording session. Thus, one CP child (CP5) was able to conclude just four blocks of trials, while only three blocks were recorded in two TDC (TD2 and TD7) and in two CPC (CP2 and CP7).

### Data Processing

In order for a trial to be included in the quantitative analysis of the oculomotor response, participant gaze had to be directed within a circle of 1° radius around the central fixation cross (in the absence of eye blinking) at the time of the target appearance in the *saccadic task* or at the appearance of the cue in the *cue-target task*.

Oculomotor responses were analyzed off-line by a custom-written Visual Basic application, developed in a Microsoft Visual Studio 2015 environment. Statistical analyses were performed in the R environment (R Core Team, 2016).

## Results

### Saccadic Task

The *saccadic task* was meant to ascertain whether CPC were able to perform visually-guided saccades with similar characteristics with respect to TDC. To this end, reaction time and spatial error (distance between target position and eye position at the end of the first saccadic movement following the target onset) were measured for simple visually-guided saccades in both groups of children.

**Figure 2:**
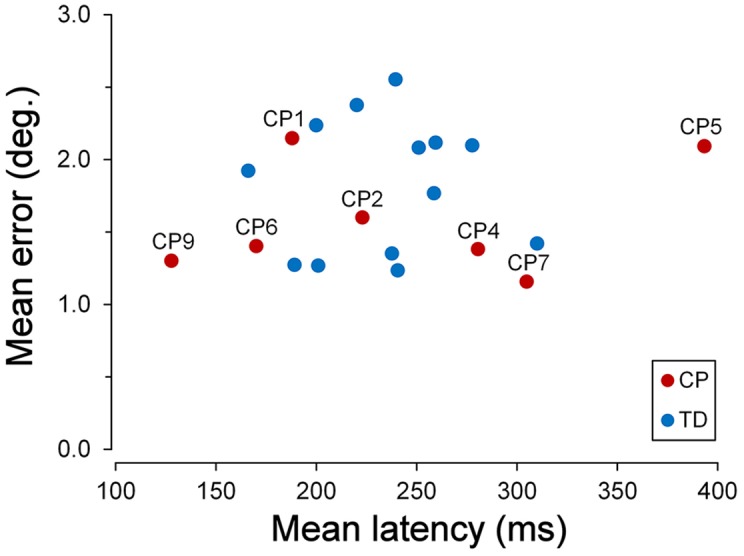
Latency and accuracy of ocular responses in the *saccadic task*. Scatter plot of the mean latencies vs. the mean errors of saccadic movements for every cerebral palsy (CP) and typically developing (TD) participant, tested in the *saccadic task*. For Children with CP (CPC), subject identifiers are shown.

The scatter plot of Figure 2 shows the mean latencies and the mean errors of saccadic movements for every CP and TD subject. The mean saccade latency across all TDC was 234.7 ms (SD ±39.14 ms), while the mean saccadic error was 1.83° (SD ±0.46°). The average values for CPC were 241.1 ms (SD ±91.14 ms) for the latency and 1.59° (SD ±0.39°) for the saccadic error. The mean values of both parameters were not statistically different for the two children groups (Wilcoxon Test, *p* > 0.39). However, the standard deviation of mean saccadic latencies was significantly larger for CPC than for TDC (*F*_(6,12)_ = 5.422; two-tailed *p* = 0.013).

This larger variability was due to the very fast and very low response speed of subjects CP9 and CP5, respectively. The mean saccadic latency of CP9 (9 years) was 127.8 ms (SD ±34.4 ms). In fact, 76% of the responses of this child were express saccades, i.e., with a latency shorter than 140 ms (Fischer and Ramsperger, 1984; Fischer and Weber, 1993). By contrast, responses of CP5 ($15\frac{1}{2}$ years) had an average latency of 385.0 ms (SD ±151.1 ms), with no express saccades. Both mean latencies fell outside the 95% confidence interval of TDC distribution (*t*_(12)_ = 2.842, *p* = 0.015 and *t*_(12)_ = 4.220, *p* = 0.001, respectively).

Moreover, latency in TDC was shorter for upward than for downward saccades, the mean intra-subject difference being 31.4 ms (SD ±35.9 ms). A paired *t*-test demonstrated that this difference was statistically significant (*t*_(12)_ = 3.153, *p* = 0.008). By contrast, the difference between upward and downward saccade mean latencies in CPC was found to be statistically non-significant (*t*_(6)_ = 2.043, *p* = 0.087).

### Cue-Target Task

#### Fixation Stability During Task Execution

Besides inhibiting a prepotent saccadic response towards the task-irrelevant visual cue, a correct execution of the *cue-target task* required the capacity of actively keeping a steady fixation, maintaining sustained attention for a long time at a specific point of the visual scene.

Fixation during trials was considered to be accurate when no eye movements larger than few degrees were made away from the central cross during the initial part of the task, or from the peripheral target once the latter was reached after the presentation of the cue-target sequence. Therefore, in our analysis, fixation accuracy measured the overall child’s ability to execute the task, independently of whether or not he/she succeeded in suppressing an eye movement towards the visual cue.

**Figure 3:**
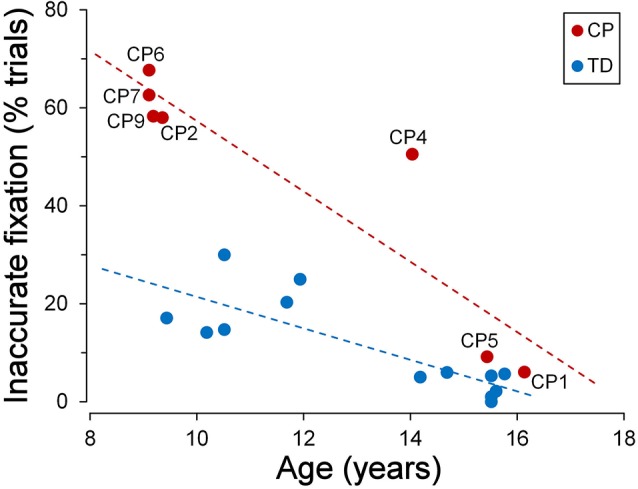
Fixation stability during the *cue-target task*. Scatter plot showing the relationship between the percentage of trials with inaccurate fixation and participant’s age. Subject identifiers are reported near CPC data points. Statistically significant regression lines (dashed) are depicted for typically developing children (TDC; blue) and CPC (red), separately.

TDC were generally quite good in keeping a steady fixation. However, we found a significant correlation between fixation accuracy and child age. The graph of Figure 3 depicts the relationship between the percentage of the trials with inaccurate fixation and the participant age, for both TDC and CPC. It can be clearly seen that older TDC (14–16 years) were very good in performing the task, as eye movements breaking the fixation periods occurred, on average, in only 3.6% of the trials, while in younger TDC (9–12 years) the mean percentage increased to 20.2%. A linear regression analysis to test the dependence of the percentage of trials with inaccurate fixation on age yielded statistically highly significant results (*β* = −0.031; *t* = −4.606; *p* < 0.001).

Figure 3 also neatly shows that younger CPC were much less accurate in task execution with respect to TDC, suggesting an impairment of executive functions. The most frequent observed incorrect behaviors were: looking towards empty areas of the visual field during task performance (sometimes even outside the computer screen), directing gaze towards “inactive” placeholders, returning to the fixation cross shortly after the eye response to the target (often with to-and-fro movements between central cross and target), “forgetting” to return to the fixation cross after the target turned off (sometimes remaining on the empty placeholder for the all duration of the next trial), or a combinations of these actions. Indeed, 62% of the trials, in the CPC below the age of 10, suffered on average of some type of error in task execution. Furthermore, these fixation inaccuracies were unlikely to depend on visual fatigue, since their frequency did not increase during a block of trials, neither towards the end of the experimental session.

Interestingly, fixation accuracy was much more accurate in the two older CPC (>15 years), at a level comparable to that of age-matched TDC. The percentage of trials with inappropriate saccadic movements was 6.1% for CP1 and 9.2% for CP5. A *t*-test to compare these values with those measured in the 7 TDC with age >14 years, yielded a statistically non-significant difference for CP1 (*t*_(6)_ = 1.081; *p* = 0.321) and a marginally significant difference in CP5 (*t*_(6)_ = 2.447; *p* = 0.050). The linear regression coefficient between percentage of trials with inaccurate fixation and age in CPC was highly significant (*β* = −0.072; *t* = −4.927; *p* = 0.004) and was twice as steep of that of TDC. Therefore, the difference between TDC and CPC in trial execution errors is large at a younger age but is virtually absent at an age of about 15.

#### Saccadic Intrusions Towards Placeholders

Saccadic intrusions towards “inactive” placeholders (i.e., when they did not exhibit any change in their luminance intensity) constituted the most common reason for fixation breakdown in both TDC and CPC.

**Figure 4:**
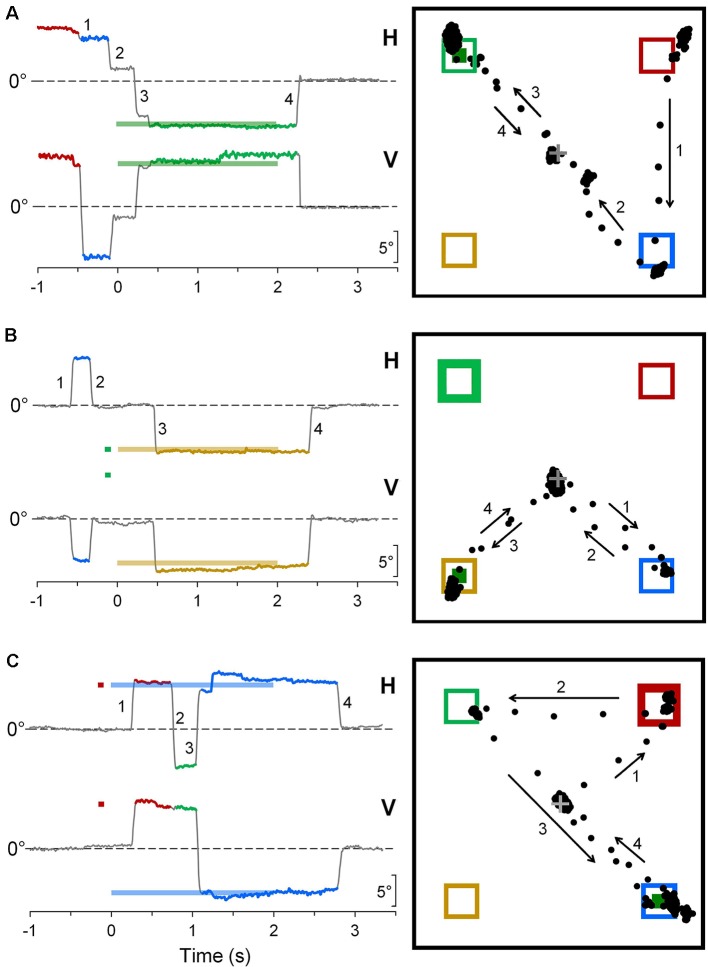
Representative recordings of saccadic intrusions towards placeholders in CPC. **(A)** A *saccadic task* trial; **(B,C)** trials during the* cue-target task*. Color codes are used to identify more easily the placeholders on which visual stimuli are presented and eye movements are directed to. On the column at the left-hand side, traces represent the time courses of horizontal (H) and vertical (V) eye movement recordings (up: rightwards and upwards direction), with respect to central fixation cross (dashed line). Bold horizontal lines, with the color corresponding to the placeholder of appearance, indicate timing and position of saccadic target and cue. Eye movement traces are also drawn with the color code corresponding to the placeholder to which gaze was directed. Insets on the right-hand column depict the projections of the line of gaze on the computer screen during the trial. Numbers and arrows mark sequence and direction of saccades, respectively, to ease the comparison between the two ways of representing the eye movements. Bold frames indicate the placeholder of cue appearance; little green squares indicate the place of occurrence of the saccadic target.

Some representative examples of saccadic intrusions in CPC are shown in Figure 4. Panel A depicts an eye movement recording during a *saccadic task* trial in subject CP6. Although instructions were to keep a steady fixation of the central cross until target onset, the subject gaze was jumping from one placeholder to another, landing in the proximity of the central cross only 110 ms before the appearance of the peripheral visual stimulus. Nevertheless, the occurrence of a subsequent correct saccadic response to the target, with a very fast reaction time of 156 ms, denotes a normal performance of visually-guided saccades. Figures 4B,C show a correct and an erroneous response, respectively, in *cue-target task* trials, in another CP subject (CP4). In Figure 4B, an eye movement to the cue was correctly inhibited, but a saccade to an “inactive” placeholder was performed during the fixation period before stimulus presentation. In Figure 4C, instead, the first saccadic response was erroneously made towards the cue with a latency of 351 ms. Gaze eventually reached the target location about 1 s after its onset, but only after the intrusion of a task-inappropriate saccade to an “inactive” placeholder.

**Figure 5:**
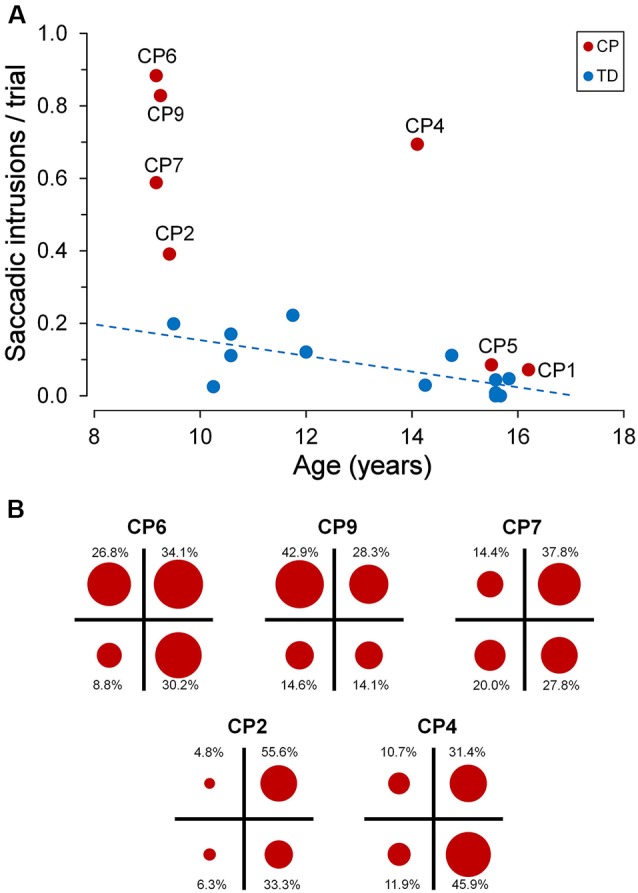
Saccadic intrusions towards “inactive” placeholders. **(A)** Scatter plot illustrating mean saccadic intrusions per trial as a function of participant age, for TDC and CPC. Subject identifiers are reported near CPC data points. The blue dashed line indicates the presence of a statistically significant linear regression for TDC data. **(B)** Distribution of saccadic intrusions among visual quadrants, made by CPC with a high rate of intrusion occurrence. In each plot, the circle areas are proportional to the relative frequency of intrusions in the corresponding quadrant (see text for details).

Figure 5A depicts the frequency of occurrence of saccadic intrusions towards placeholders during the entire experimental session (measured as mean intrusions per trial) as a function of age, for both TDC and CPC. In TDC, they occurred on average about once every 12 trials (0.084 intrusions/trial, SD ±0.077). Moreover, as for fixation accuracy, also the frequency of saccadic intrusions to placeholders had an inverse linear relationship with age, reaching an almost perfect inhibition of these incorrect gaze shifts at the age of about 15 years. The regression analysis on TDC data yielded a statistically significant regression coefficient (*β* = −0.020; *t* = −2.742; *p* = 0.019).

By contrast, younger CPC made much more frequent gaze movements towards “inactive” placeholders than TDC, often performing several saccadic intrusions within the same trial. The number of intrusions per trial ranged from 0.391 (CP2) to 0.884 (CP6), falling largely outside the two-tailed 95% confidence interval of the distribution found in the TDC population. Interestingly, saccadic intrusions were scanty in the two older CPC (CP1 and CP5), with a frequency of occurrence very similar to that of age-matched TDC (15–16 years). Not surprisingly, these two subjects were also very good in maintaining a steady fixation during the execution of the task (see Figure 3).

The execution of saccadic intrusions towards placeholders was of particular interest in CPC since most subjects showed a quadrant preponderance in their occurrence. Figure 5B represents the distribution of saccadic intrusions among visual quadrants, in each of the five CPC with a high rate of intrusion occurrence. For each participant, the total area of the four circles is proportional to the overall frequency of occurrence per trial. Moreover, the area of each circle reflects the proportion of intrusions (whose percent value is reported nearby) directed to the placeholder located in the corresponding visual quadrant. In all subjects shown in Figure 5B, a *χ*^2^ test indicates that the observed frequencies differed significantly (*p* ≤ 0.001) from those expected if the distribution of saccadic intrusions were the same among the four quadrants. Subjects CP2 and CP4 showed a neat left-right asymmetry, with a marked preponderance of saccadic intrusions to the right visual hemifield. By contrast, in subject CP9 the visual field preponderance was towards the upper quadrants. Finally, in CP6 and CP7 the distribution of saccadic intrusions was clearly non-uniform, with an obvious lower frequency of occurrence towards the lower-left and upper-left quadrants, respectively.

#### Responses to Cue

A goal of the cue-target paradigm was to ascertain the operation of the inhibitory control in CPC in presence of a stimulus-driven capture of attention: the task-irrelevant cue is a prepotent attentional stimulus, eliciting a foveating saccade that must be suppressed. For the purpose of this analysis, we considered a saccadic response as erroneously elicited by the cue appearance: (1) an *invalid* trial in which the first saccade following the presentation of the cue-target pair of stimuli was directed towards the cue; and (2) a *valid* trial in which the eye movement was directed towards the target with a latency shorter than 90 ms. In fact, below this very short latency, it is safe to assume that the response was driven by the luminance change of the placeholder (cue), rather than by the presentation of the target.

**Figure 6:**
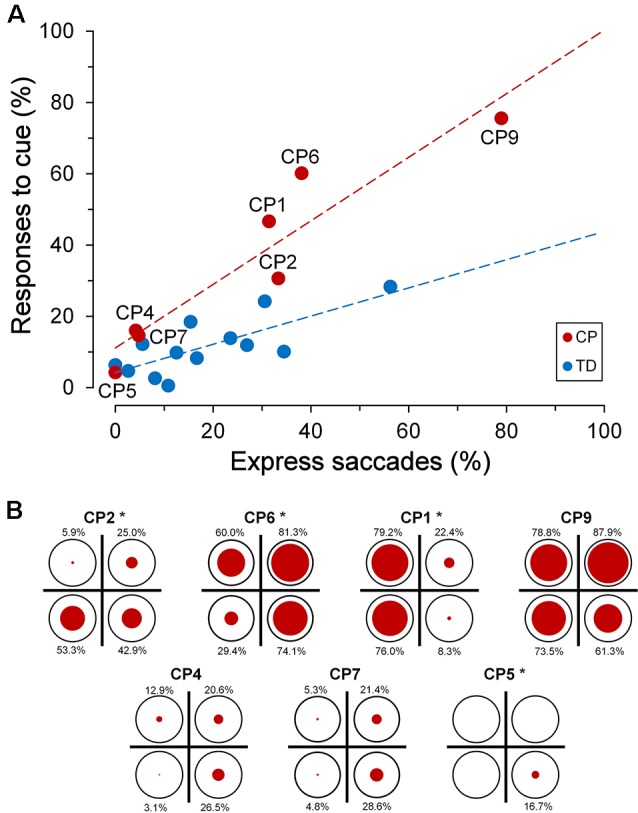
Inhibitory control of reflexive saccades in the *cue-target task*. **(A)** Scatter plot depicting the percentage of eye responses towards the cue as a function of the percentage of express saccades made by each subject. Subject identifiers are reported near CPC data points. The red dashed line indicates the presence of a statistically significant linear regression for CPC data. **(B)** Distribution of responses towards the cue among visual quadrants for each CP subject. In each plot, the size of the red circles reflects the percentage of responses (whose value is reported nearby), with respect to the number of trials in which the cue fell in the corresponding quadrant (indicated as reference by a black circumference). Asterisks near the subject identifiers indicate a non-random distribution of cue-directed saccades.

Interestingly, the ability to suppress an automatic response towards the cue was markedly affected by the subject’s “response readiness” in performing visually-guided saccades, as measured by the rate of the express saccades recorded during the *saccadic task*. Express saccades are saccades that are characterized by an extremely short latency (Fischer and Ramsperger, 1984; Fischer and Weber, 1993). It has been reported that a relatively high number of express saccades determines a reduced ability to suppress reflexive saccades (Fischer et al., 1997), suggesting the presence of a poorly developed fixation system. In this article, a saccade has been defined as “express” when its latency falls within the range of 90–140 ms. In agreement with literature data, we found, in both TDC and CPC, a statistically significant linear correlation between the percentage of eye responses elicited by the cue and the number of express saccades made by each subject (Figure 6A).

A linear regression analysis was conducted to compare the relationship of the percentage of responses to cue to the rate of express saccades, for each group of children. The analysis yielded a highly significant correlation between the percentage of responses to the cue and the express saccade frequency, for both TDC (*β* = 0.396, *R*^2^ = 0.577, *p* = 0.003) and CPC (*β* = 0.892, *R*^2^ = 0.886, *p* = 0.002). A significant interaction in the relationship was found for the two groups of children (*F*_(1,16)_ = 8.73, *p* = 0.009), indicating a statistically significant difference between the regression coefficients. Finally, the two groups of children did not show a significant difference in the rate of occurrence of expressed saccades (Wilcoxon test, *p* = 0.663), suggesting that CP does not determine *per se* a variation in the tendency to make saccades with a very short latency. Notwithstanding the lack of a statistical difference between the population means, it is worth noting that subject CP9 made an unusually high number of express saccades (78.9%), yielding an extremely short overall saccadic mean latency (127.8 ms).

To summarize, in both TD and CP children, the ability to suppress an eye movement towards a prepotent attentional stimulus strongly depends, in an inverse manner, on the individual leaning to make saccadic responses with very short latencies. Thus, CPC that idiosyncratically make most saccades with a regular latency are as successful as TDC to inhibit gaze shifts towards task-irrelevant captures of attention. An impairment of the inhibitory control of reflexive saccades with respect to TDC manifests only in CPC who have the tendency to make visually-guided saccades with very short reaction times: the highest the rate of express saccades, the worst is an effective suppression of a response to the cue stimulus.

Alike to the observed spatial preponderance for the saccadic intrusions towards placeholder, also the ability of most CPC to suppress a saccade towards the cue was not equally compromised in the various quadrants of the visual field. Figure 6B depicts, for every CP subject, the distribution among quadrants of the responses to cue. The size of the red circles reflects the percentage of responses (whose value is reported nearby), with respect to the number of trials in which the cue fell in the corresponding quadrant (indicated as reference by a black circumference). Asterisks near the subject identifiers indicate that the observed percentages of responses to cue differed significantly from those expected if they were equally distributed in all quadrants (*χ*^2^ test at *p* < 0.05 level). Specifically, differences in distribution among quadrants were found to be statistically highly significant (*p* < 0.001) in CP1, CP5 and CP6, while in CP2 the *χ*^2^ test yielded *p* = 0.021.

By taking into account the four CPC who manifested the biggest impairment in suppressing the responses to the cue, one can notice that inhibitory control was indeed normal or near-to-normal in some quadrants, but was highly defective in others. Thus, in CP1 inhibitory control was almost completely lost in the left visual hemifield, but was very similar to that of the TDC population on the right hemifield. In CP2, the impairment affected only the lower hemifield, while CP6 failed in suppressing cue-directed saccades in the whole visual field, except the lower-left quadrant. Finally, inhibitory control was almost completely lost in the whole visual field in CP9, with no statistically significant differences among quadrants (*χ*^2^ = 6.394; *p* = 0.094).

Interestingly, the quadrants in which a subject made more frequently saccadic intrusions to placeholders did not correspond, in general, to those in which responses to cue were prevailing. For instance, saccadic intrusions were almost absent in CP1, but saccades to the cue occurred in the large majority of trials in the left hemifield. Subject CP2 made saccadic intrusions mostly in the right hemifield but showed a preponderance of responses to the cue in the lower quadrants. Conversely, a good spatial correspondence of saccadic intrusions and responses to the cue was present in subject CP6.

#### Effect of Cue Validity on Saccade Latency

In the *cue-target task*, the participant had to perform a saccade to the peripheral target, after a visual cue was flashed a short time interval (150 ms) before the target onset, either at the same (valid cue) or at a different (invalid cue) spatial position. Under these conditions, saccadic responses were expected to be faster when the target occurred at cued, relative to uncued, locations.

Our cueing paradigm proved to be very effective in inducing faster responses in TDC when the cue occurred at the same location as the saccade target. We performed a two-way repeated-measure ANOVA, with “*validity*” and “*target quadrant*” as grouping factors, on the within-subject mean response latencies, computed from all correctly performed trials. Analysis yielded highly significant principal effects for both factors, with a non-significant interaction. Cue validity accounted for an average increase of 49.3 ms in saccade latency of invalid with respect to valid trials (*F*_(1,84)_ = 66.293, *p* < 0.0001). Mean latency across subjects was 211.0 ms and 260.3 ms for valid and invalid trials, respectively. Also the “*target quadrant*” principal effect was highly significant (*F*_(3,84)_ = 9.391, *p* < 0.0001). This effect was largely accounted for by the fact that response latencies towards the upper visual hemifield (221.7 ms, SD ±88.3 ms) were faster than those towards the lower hemifield (249.6 ms, SD ±94.2 ms). Accordingly, a two-way repeated-measure ANOVA yielded very significant “*up-down*” (*F*_(1,36)_ = 15.141, *p* = 0.0004) and “*validity*” (*F*_(1,36)_ = 47.298, *p* < 0.0001) principal effects, with a non-significant interaction.

The effect of cue validity on saccade latency was more difficult to ascertain in CPC as a population, because of a non-homogeneous behavior in the *cue-target task* relative to the visual quadrants and the scantiness of usable responses in some subjects. In fact, as described above, many trials had to be discarded for this type of analysis, either for the preponderance of cue-directed responses or for the presence of saccadic intrusions or other fixation inaccuracies. Nevertheless, a two-way repeated-measure ANOVA on the within-subject mean response latencies revealed a significant effect of the factor “*validity*” (*F*_(1,45)_ = 4.427, *p* = 0.041) and a non-significant effect of the factor “*target quadrant*” (*F*_(3,45)_ = 1.167, *p* = 0.333). The reason for the low level of significance of the “*validity*” effect is possibly to be ascribed to the large statistical variability of the data, due to the reduced number of available trials per quadrant in some subjects. In any case, the mean intra-subject increase in response latency for the invalid trials with respect to the valid ones was 57.7 ms, that is, quite similar to that found in the TDC population.

## Discussion

This article investigated attentional and inhibitory control in CPC, with normal IQ or mild cognitive impairment, by using oculomotor tasks. This approach was meant to overcome the difficulties arising from postural and limb movement disabilities when tests are based on manual responses. Furthermore, the intimate relationship between eye movements and spatial attention constitutes a vantage point to discern deviant attention engagements, increased distractibility or deficits in discarding irrelevant sensory stimuli to the ongoing task.

The present study extends recent research demonstrating that CPC might show significant impairments in inhibitory control (Christ et al., 2003) and of attentional and executive skills (Bottcher et al., 2010; Bodimeade et al., 2013). Indeed, our results indicate that CPC show severe deficits in maintaining sustained attention and complying with instructions of the oculomotor task. Furthermore, inhibitory control appears to be significantly impaired. Patients show great difficulties in suppressing saccades not only to the cue stimuli but also to “inactive” placeholders, which represent powerful attentional attractors that must be covertly attended during task execution. Altogether, results provide evidence that CPC often manifests significant executive impairments, even in the presence of normal or mildly impaired intelligence.

These findings have relevant implications from a clinical and rehabilitative viewpoint. It is widely accepted that the maturation of attentional control and of the ability to suppress responses to stimuli, that are irrelevant or conflicting with the ongoing task, is essential for the development of cognitive abilities through childhood and adolescence (Dempster, 1993; Anderson et al., 2002). Accordingly, a number of studies have shown that CPC often manifests specific learning disabilities, lower academic performance and problems in emotional and social relationships (Frampton et al., 1998; Nadeau and Tessier, 2006; Parkes et al., 2009; Whittingham et al., 2010).

### Saccadic Inhibitory Control After Stimulus-Driven Captures of Attention

There is a large body of evidence that spatial attention and saccade programming are driven by overlapping neural mechanisms (Rizzolatti et al., 1987; Awh et al., 2006). Because of the drop in visual acuity with increasing retinal eccentricity, a saccade is the normal motor response to bring into the fovea a salient visual object for a better perceptual processing. However, the system has evolved to make covert shifts of attention (i.e., without the overt deployment of an eye movement), whenever the motor response is inadequate to the behavioral goal. Because of the tight link between selective attention and saccade planning, a covert shift of attention relays on a successful inhibition of the programmed eye movement. Our data show that a deficit in the saccadic inhibitory control following an attentive engagement is a common outcome of CP.

Capture of attention can be driven by two distinct mechanisms, controlled by two partially segregated neural systems (Corbetta and Shulman, 2002). Stimulus-driven (*bottom-up*) capture of attention takes place at the occurrence of an unexpected or salient stimulus. By doing so, these events gain high priority over brain activity, in order to advantage the perceptual processing of the novel stimulus. By contrast, goal-directed (*top-down*) shifts of selective attention are controlled by cognitive factors, such as expectancies, task-related instructions and behavioral goals. In the *cue-target task* of this study, both stimulus-driven and goal-directed captures of attention are present.

Cue stimuli in the *cue-target task*, although uninformative, represent novel events that increase the saliency of a specific spatial location and generate a stimulus-driven capture of attention. The attentive engagement by the cue event is demonstrated by a well-known spatial priming effect, which determines faster responses and an enhanced stimulus detection when a target stimulus occurs at the same location within a time interval shorter than 200 ms (Posner et al., 1984; Fecteau et al., 2005; as opposed to the phenomenon called “inhibition of return” occurring at longer time intervals; Posner and Cohen, 1984; Fecteau et al., 2005). At the cue-target onset asynchrony employed in this study of 150 ms, therefore, valid cues are expected to have a facilitatory effect on the latency of the eye movement towards the target. Accordingly, a significant decrease of the saccadic reaction time in the valid-cue trials, with respect to the invalid ones, is observed in both the TDC and CPC populations. This finding demonstrates that cue stimuli in our experimental protocol are effective in inducing a *bottom-up* engagement of spatial attention and that the basic mechanisms of stimulus-driven capture of attention are preserved in our sample of CPC. However, data show that many CPC have much greater difficulties, with respect to TDC, in suppressing a saccadic response towards the task-irrelevant visual cues. By comparison, TDC on average fail to inhibit an eye movement to the cue only in about 12% of the trials. Instead, there are quadrants of the visual field in which 4 out of 7 CPC make a saccade to the cue in more than 50% of the responses, reaching in some subjects percentages higher than 75–80%. The correct suppression in some quadrants of the responses to the cue in all CPC (except CP9), to a level comparable to TDC performance, demonstrates that, in spite of the presence of a mild cognitive impairment in some participants, the deficit in saccadic inhibitory control cannot be ascribed to a poor understanding of the instructions of the *cue-target task*. In fact, if performance errors were due to a lack of comprehension of the task, we would expect a uniform spatial distribution of the responses to the cue in the visual field. In addition, the number of execution errors does not seem to be related to the VIQ or FIQ scores of the participant (see Table 1).

Interestingly, in both TDC and CPC, the ability to inhibit stimulus-driven saccades towards the cue is inversely proportional to the frequency of express saccades made by the subject during a visually-guided saccade task. Express saccades are generally produced in low numbers, especially with an overlap paradigm as in the *saccadic task* of this study, i.e., when the fixation point remains visible during the presentation of the saccade target. According to Fischer et al. (1997), the rate of express saccades in the overlap condition is <20% in young subjects (less than 20 years old). Munoz et al. (1998), instead, reports a percentage range of 0–35% (mean 9.3%) in children with ages between 5 and 8.

In the present study, the rate of occurrence of express saccades does not show a statistical difference between TD and CP children. The percentage is <40% for the majority of participants, independently of age. It should be noted, however, that two children make an extraordinarily high number of express saccades: 56.3% (TD child) and 78.9% (CP9). The higher rate of express saccades in this study, with respect to that reported in the literature, may find a plausible explanation in the difference in the experimental protocol. In our paradigm, placeholders are always present, possibly exerting a priming effect on the locations where the saccade target will appear. In addition, a warning acoustic tone occurs at a fixed interval from the target onset, enhancing the subject readiness to respond to the visual stimulus. Finally, a note on the two children who perform a very high number of express saccades. One could hypothesize that, independently of being affected by CP, these subjects belong to the minority of individuals, known as “express saccade makers,” who produce unusually high numbers of express saccades in the overlap paradigm (Biscaldi et al., 1996; Cavegn and Biscaldi, 1996). This condition has been proposed to result from a poor development of the fixation system and is associated with a marked difficulty to suppress reflexive saccades and with a reduced voluntary control over saccade generation. Therefore, based on our data, it is not possible to conclude that the observed tendency in some CPC to make a high number of express saccades is a consequence of the early brain lesion.

The relationship between the percentage of responses to the cue and express saccade rate is also in full agreement with literature data. In fact, it has been reported that the capability of suppressing reflexive saccades in an antisaccade task (subject has to inhibit a saccade towards a visual stimulus and move his gaze to its mirror location; Hallett, 1978) or in a memory-guided saccade paradigm (execution of a delayed eye movement towards the spatial location of a briefly presented visual stimulus) is reduced as a function of the rate of express saccades that each subject makes in a visually-guided saccade task (Fischer et al., 1997; Munoz et al., 1998).

In this context, however, the most relevant result is that, at equal percentages of express saccade execution, CPC make more saccadic responses to the cue than TDC. This represents a clear demonstration of an impairment of the saccadic inhibitory control in the presence of a stimulus-driven capture of attention. Interestingly, this difficulty in suppressing task-irrelevant, reflexive saccades becomes manifest only in CPC who tend to make visually-guided saccadic responses with very low latencies. Patients normally performing saccades with regular/long reaction times are able to inhibit responses to the cue stimuli as efficiently as TDC.

### Cognitive Control of Oculomotor Behavior

Placeholders in our experimental setup constitute powerful goal-directed attentional attractors, inasmuch as they are locations in which behaviorally relevant sensory stimuli are expected to occur. They are always present throughout the recording session, representing areas of interest that are covertly attended while the subject is looking to the detection of the target onset. The occurrence of a *top-down* attentive engagement (together with an associated oculomotor program) is indicated by the occasional presence, even in TDC, of escape saccades during the periods of visual fixation, bringing temporarily the subject’s gaze on an “inactive” placeholder. This interpretation is also supported by the notion that expectancy can induce a sustained neuronal activity in the fronto-parietal network subserving selective attention, even in the absence of a novel visual stimulus (Kastner et al., 1999). It is noteworthy that, in our group of TDC, the inhibitory control of inappropriate saccades towards goal-directed attentional attractors improves with age, attaining a very high level of performance at 15–16 years (Figure 5A).

As for stimulus-driven shifts of attention, our data show that saccadic inhibitory control in CPC is impaired also with goal-directed attentive engagements. This is especially evident in younger children, who make many more saccadic intrusions to placeholders than TDC. Also in this case, performance appears to improve with age. Although the low number of subjects does not allow drawing definitive conclusions, 15–16-year-old CPC exhibit an almost perfect ability to suppress saccades towards placeholders, to a degree that is virtually identical to that shown by age-matched TDC.

To summarize, the present study supports that an outcome of early brain lesions is a deficit in the saccadic inhibitory control in the presence of both stimulus-driven and goal-directed captures of attention. This impairment does not affect in equal manner the whole visual field but shows a marked spatial selectivity in each individual subject. Furthermore, the quadrant spatial preponderance of this deficit is often different for *bottom-up* and *top-down* attentive engagements. This result can find an explanation in the ordered spatial topography of the multiple neural representations of the attentive map (e.g., Fecteau and Munoz, 2006) and the fact that partially segregated brain networks control the two types of attention mechanisms (Corbetta and Shulman, 2002).

### Maturation Timing of a Stable Fixation

Several studies in the literature investigated in TDC the maturation of the ability to suppress context-inappropriate saccades and to maintain a stable fixation for a prolonged time-span. There is a wide consensus that these abilities, like many other executive skills (e.g., Anderson, 2002), attain an adult level of performance by the age of 15–20 years (Fischer et al., 1997; Munoz et al., 1998). For instance, the rate of directional errors of saccadic responses in the antisaccade task decreases from about 50% to 10% between age 8–9 and 15–17 years, according to Munoz et al. (1998), and from 60% to 22% between age of 9 and 15 years, according to Fischer et al. (1997). The ability to maintain a stable central fixation, in the presence of distracting peripheral visual stimuli, also markedly improves between 9 and 10 years of age (Paus et al., 1990). However, 10-year-old children are still unable to suppress verbally forbidden saccades in about 40% of the trials, a rate well above adult level. Saavedra et al. (2009) reported similar results regarding the ability to maintain central fixation at the appearance of a peripheral target. In that study, the rate of fixation breakdowns in CPC decreases from 80% at the age of 6–9 to about 40% at the age of 11–16, against an about 20% of errors in age-matched TDC.

Our data in TDC are in good agreement with the literature. Task instructions were to keep a steady fixation either on the central fixation cross or on the target, depending on the phase of the task paradigm. Between the age of 9 and 12, the average percentage of trials with fixation inaccuracies is about 20%. More importantly, an inverse correlation is present between rate of fixation breakdowns and age, with the achievement of a high level of fixation accuracy at 15–16 years. Therefore, there is a clear evidence that, within the age-span of this study, the executive abilities required to perform the oculomotor tasks of this study are still undergoing a process of maturation.

The ability to maintain a stable fixation is far worse in younger CPC. If we take into account children below the age of 10, one or more fixation breakdowns occur in the large majority of trials, as described in more detail in the section “Fixation Stability During Task Execution” of the “Results” section. It should be noted that visual field is normal in younger CPC. Therefore, the more frequent execution of saccades cannot be ascribed to a compensatory strategy in the presence of a visual field constriction. It should be stressed, however, that both saccadic intrusions towards placeholders and fixation inaccuracies decrease with age more steeply in CPC than in TDC. This trend leads to a reduction over time of the gap in performance, until a similar executive high level is attained by the age of about 15 years. This time course of performance improvement is very similar to that described by White and Christ (2005), although in a different context of executive abilities.

### Concluding Remarks

Our results provide compelling evidence that early brain injuries determine in childhood deficits of some executive skills in the oculomotor behavior, which recover to virtually normal level during adolescence. A development delay of executive abilities is not an adequate explanation for this observation, since: (1) also TDC exhibit, in the age-span of this study, an improvement of the saccadic inhibitory control and of fixation accuracy, although showing a considerably higher level of performance with respect to CPC; and (2) both TD and CP children attain the same high level of performance at about 15–16 years. Therefore, it looks more a matter of a greater incompetence of immature executive skills in CPC, rather than a delay in the attainment of abilities that are normally achieved at an earlier age.

We can make some speculations about the neural substrate underlying these observations. It is widely accepted that the development of the cognitive control is bound to the maturation of the frontal lobes and of basal ganglia thalamo-cortical circuits (Krasnegor et al., 1997; Casey et al., 2001), which typically occurs during the second decade of life. While the dependency of executive processes from the prefrontal cortex (PFC) in the adult brain is undisputed, recent studies have shown that the integrity of the entire brain is essential in childhood for typical executive performance (Jacobs et al., 2011; Long et al., 2011). During the maturation process, executive functions are not yet localized in the PFC but have a more diffuse neural representation. Accordingly, functional MRI studies have shown that children recruit different brain regions from adults in executive tasks involving response inhibition or interference suppression (Luna et al., 2001; Bunge et al., 2002) and early focal lesions induce similar patterns of executive deficits, regardless of their localization (Jacobs et al., 2011). Furthermore, contrary to adults and to 14–17-year-old adolescents (Luna et al., 2001), activation of PFC does not occur in younger children, in whom executive abilities are bound to an activation of more posterior cortical areas. One could then surmise that the deficits occurring at a younger age, following an early brain injury, could result from a greater difficulty of extra-frontal regions in providing for executive functions, which afterward will become a main prerogative of the frontal lobes.

Along this way of reasoning, it is possible to hypothesize that the maturation process of PFC circuits, and consequently the unfolding of the related cognitive abilities, do not have a very dissimilar time course in TD and CP children, leading to an alike executive competence at about the age of 15–16 years. By contrast, the lower level of performance we observed during the earlier development period (in abilities such as suppressing saccades towards attentive stimuli, focusing attention for extended periods and exerting a cognitive control of oculomotor responses) mainly occurs in the time epoch in which executive skills seem to depend on the involvement of extra-frontal regions, conceivably through alternative executive strategies. A possible explanation of the larger executive deficits in younger CPC is that early brain lesions make this functional substitution process more difficult, determining a worse capacity to control behavior from cognitive factors.

Obviously, this condition should not be considered as a temporary situation that produces only transitory effects. The first decades of life constitute a critical period for the cognitive development and the achievement of behavioral competence. An insufficient ability to discard task-irrelevant sensory stimuli, to engage sustained attention or to inhibit a prepotent motor response, may represent a relevant factor facilitating the emergence of learning disorders and social difficulties, frequently affecting CP children and adolescents (Frampton et al., 1998; Bottcher et al., 2010; Whittingham et al., 2014).

## Data Availability Statement

The datasets generated for this study are available on request to the corresponding author.

## Ethics Statement

The studies involving human participants were reviewed and approved by Ethics Committee of “ASST Spedali Civili” of Brescia, Italy (protocol. N. 1324, 08/04/2013). Written informed consent to participate in this study was provided by the participants’ legal guardian/next of kin.

## Author Contributions

CM, LF, EF and MB contributed to the conception and design of the study. EF, JG, SM and LT performed participant recruitment. LF, JG, SM and LT performed data collection and eye movement recordings. CM and LF performed the data analysis. CM wrote the first draft of the manuscript. All authors contributed to manuscript revision, read and approved the submitted version.

## Conflict of Interest

The authors declare that the research was conducted in the absence of any commercial or financial relationships that could be construed as a potential conflict of interest.

## Footnotes

^1^www.neurobs.com

^2^idk.fh-joanneum.at/2018/04/22/visionspace
